# Comparison on the status of vitamin A in 6- to 13- year-old children between 2002 and 2012 in China

**DOI:** 10.1186/s12937-016-0170-0

**Published:** 2016-05-04

**Authors:** Chun Yang, Jing Chen, Ning Guo, Zhen Liu, Chunfeng Yun, Yajie Li, Jianhua Piao, Xiaoguang Yang

**Affiliations:** 1 Key Laboratory of Trace Element Nutrition, National Health and Family Planning Commission of the people’s Republic of China, Department of Trace Element Nutrition, National Institute for Nutrition and Health, Chinese Center for Disease Control and Prevention, Room 236, Nanwei Road No.29, Xicheng District, Beijing, 100050 China; 2 Jinnan District Center for Disease Control and Prevention of Tianjin, Tianjin, 300350 China

**Keywords:** Vitamin A status, Children, China

## Abstract

**Background:**

Vitamin A deficiency is recognized as a major public-health nutrition issue in the developing countries. Limited hospital sources and sample sizes are available in most of the existing studies associated with healthy school-age children. The aim of this study was to compare vitamin A status of 6- to 13-year-old healthy children in China between 2002 and 2012.

**Methods:**

According to China National Nutrition and Health Survey 2002 (CHNNS2002) and CHNNS2010-2013, we choose 6- to 13-year-old children as the research object. We measure the serum retinol concentrations of the children using high performance liquid chromatography (HPLC). The children were divided into two groups: 6- to 9-year-old and 10- to 13-year-old. The areas were divided into urban and rural area.

**Results:**

Total number of the children (6- to 13-year old) was 8170 in 2002 survey, and it was 6016 in 2012 survey. In 2012, the vitamin A level of the children was higher than that in 2002 (t = 39.26, *p* = 0.000). The level of vitamin A in 10- to 13-year-old group was higher than that in 6- to 9-year-old group across areas and genders between 2002 and 2012. There was no difference on the incidence of vitamin A deficiency in all the children between 2002 and 2012, but in 2012 the incidence of vitamin A deficiency in the urban children was higher than that in 2002 (*x* ^2^ = 45.456,*p* = 0.000). The incidence of vitamin A deficiency in 10- to 13-year-old group was lower than that in 10- to 13-year-old group across areas and genders between 2002 and 2012. In 2012, the incidence of marginal vitamin A deficiency in the children was lower than that in 2002 (*x* ^2^ = 861.604, *p* = 0.000). Similar phenomena were also found in across area groups. The incidence of marginal vitamin A deficiency in 10- to 13-year-old group was lower than that in 6- to 9-year-old group across areas and genders between 2002 and 2012. There was no difference in vitamin A status of the children across the area, gender and age groups between 2002 and 2012.

**Conclusion:**

Vitamin A nutritional status of the children in 2012 has been significantly improved compared with that in 2002. However, vitamin A deficiency was still a moderate public health problem in Chinese children, especially in younger school-age children. Consequently, controlling the incidence of vitamin a deficiency is imperative for promoting Chinese children’s health.

## Background

Vitamin A is an essential nutrient needed in small amounts for the normal functioning of the visual system, and maintenance of cell function for growth, epithelial integrity, red blood cell production, immunity and reproduction [1]. The World Health Organization (WHO) defines vitamin A deficiency (VAD) as tissue concentrations of vitamin A low enough to have adverse health consequences, even if there is no evidence of clinical deficiency [2]. Serum retinol concentration is a reliable indicator of an individual’s vitamin A status [3].

VAD has been recognized as a major public-health nutrition issue in developing countries [1]. According to WHO, serum retinol concentrations are classified as ≥1.05 μmol/L for normal, 0.70–1.05 μmol/L for marginal, and <0.70 μmol/l for deficient, respectively [4]. The degree of severity of VAD is defined as mild, moderate and severe: the prevalence of marginal or deficient serum/plasma concentrations is 2–10 %, if prevalence is 10–20 % and if prevalence is over 20 % [1]. Previous reports have made an effort to identify VAD in the children at the age of 5 to 15 years in Southeast Asia. It is suggested that 23 % of the population (83 million) in this age range had lower serum retinol concentrations than 0.7 mmol/L [5], so VAD is considered as a serious public health problem in Southeast Asia. There are some risk factors associated with VAD, such as gender, age, weight, socio-demographic characteristics, socio-economic status of the family, nutritional habits of the children, recent infections, and maternal education and so on [6–8]. However, little data are available on the status of VAD of 6- to 13-year-old children in China. Therefore, there is an urgent need to illustrate the prevalence of VAD in healthy school-age children in China.

This study was designed to compare vitamin A status of 6- to 13-year-old children between 2002 with 2012 in China, and to provide information regarding whether the VAD constitute a public health problem. The findings from the current study will contribute to provide means to combat VAD.

## Methods

### Study participants

The survey was conducted from August to December in 2002 with a well-designed stratified multistage cluster sampling method, and covered all 31 provinces, autonomous regions, and municipalities directly under the central government throughout China (except Taiwan, Hong Kong, and Macao). The country was divided into 6 strata (large cities, small to medium cities, class 1 rural areas, class 2 rural areas, class 3 rural areas, and class 4 rural areas) based on geographic characteristics, economy and social development, according to the China National Bureau of Statistics and China Ministry of Health Statistics. The first stage of sampling involved the random selection of 22 districts (urban) or counties (rural) from each of the 6 strata. The second stage involved the random selection of 3 neighborhoods (urban) or townships (rural) from each of the selected districts/counties. From each of the neighborhoods or townships, 2 residential committees (urban) or villages (rural) were randomly selected; 90 households were randomly sampled from each village 6 [9]. A total of 132 monitoring sites were included in the analysis, and 6 children (6- to 13- year old) were randomly selected at each site according to age and sex.

The survey in 2012 from nationally representative cross-sectional study was conducted by Chinese Center for Disease Control and Prevention to assess the health and nutrition of Chinese civilians. It covered all 31 provinces, autonomous regions, and municipalities directly under the central government throughout China (except Taiwan, Hong Kong, and Macao). A stratified multistage probability sampling design was used for the selection of participants. The country was divided into four strata (large cities, small to medium cities, general rural areas and poor rural areas) by their economic characteristics and social development. A total of 150 monitoring sites were included in the analysis, and 5 children (6- to 13- year old) were randomly selected at each site according to age and sex.

Under the leadership of the Disease Prevention and Control Bureau of Chinese Ministry of Health, National institute of nutrition and health, Chinese center for disease control and prevention was responsible for the implementation of the national level training work (grade 1), which for the provinces (autonomous regions/municipalities directly under the central government throughout China) project personnel the spot investigation and monitoring technology backbone technical training method and laboratory operation. Provincial training plan by the project team though national organizations at the provincial level training (grade 1), which to the provincial (autonomous regions/municipalities directly under the central government throughout China) all professional and technical training for staff involved in monitoring, training qualified rear can participate in the monitoring study.

The study was divided into two groups by age: 6- to 9-year-old and 10- to13-year-old. In 2002, we considered large cities, small to medium cities as urban areas, and class 1 rural area, class 2 rural areas, class 3 rural areas, and class 4 rural areas as rural areas. In 2012, we considered large cities, small to medium cities as urban areas, and general rural areas and poor rural areas as rural areas.

Ethics approval was obtained from the Ethics Committee of China Centre for Disease Control. All participants gave informed consent.

### Blood samples and biochemical measurement

Blood samples (about 4 mL) of each subject were collected by venipuncture from the antecubital vein before breakfast. The blood was centrifuged at 1500 × g for 10 min at 4 °C and then separated immediately. The centrifuged serum samples at least 500 μL were transported to the laboratory away from light and stored at -80 °C. The concentration of serum retinol was determined by using high-performance liquid chromatographic apparatus (HPLC) (Waters 2487UV detector USA) [10]. Retinol was extracted with hexane after deproteinization with ethanol containing retinyl acetate as the internal standard and evaporated to dryness with nitrogen gas. The residual materials were dissolved in 0.2 mL ethanol. The mobile phase which was a methanol: DH2O mixture (96: 4). A portion (10 μL) of the sample was injected into a column (3.9 · 150 mm; Symmetry Shield RPl8 Waters Breeze, Milford, MA, USA) installed with a HPLC (Waters 1525 Binary HPLC Pump, Waters Breeze). All procedures were performed in a dark room to protect the serum from light. The concentration of retinol was determined with a spectrophotometer (Waters 2487 Dual l Absorbance Detector, Waters Breeze) at 325 nm. Duplicate analyses were performed on one-tenth of the samples and the estimated variability was 0.02 μmol/L. The experienced examiners measured all biochemical indices.

### Statistical methods

SAS version 9.2 (SAS Institute, Inc, Cary, North Carolina) was used for all analyses. Continuous variables were presented as mean ± SD while categorical variables were presented as percentage rate. Independent-Samples *T* test were used to compare continuous variables data and the percentage rates were assessed with chi-square test. A value of *p* < 0.05 was considered statistically significant.

## Results

In 2002 survey, total number of the children (6- to 13-year-old) was 8170. The numbers of boys and girls were 4038 and 4132, respectively. In 2012 survey, total number of the children (6- to 13-year-old) was 6016. The numbers of boys and girls were 3059 and 2957, respectively. The specific numbers of children in our study were shown in Table 1.

**Table 1:** The number of 6-13 year old children at 2002 and 2012

Age group (year)	2002				2012			
	Urban		Rural		Urban		Rural	
	Boys	Girls	Boys	Girls	Boys	Girls	Boys	Girls
6–9	474	479	1872	1796	714	691	974	959
10–13	341	353	1445	1410	511	548	794	759
Total	1647		6523		2530		3486	
	8170				6016			

In 2012, vitamin A level of all the children was higher than that in 2002 (t = 39.26, *p* = 0.000). Similar phenomena were also found in different area groups. The level of vitamin A was no difference between boys and girls across the area and age groups. The level of vitamin A in 10- to 13-year-old group was higher than that in 6- to 9-year-old group across areas and genders between 2002 and 2012 (Table 2). There was no difference on the incidence of vitamin A deficiency in all the children between 2002 and 2012, but in 2012 the incidence of vitamin A deficiency in the urban children was higher than that in 2002 (*x* ^2^ = 45.456, *p* = 0.000). There was also no difference on the VAD between boys and girls across area and age. The VAD of in 10- to 13-year-old group was lower than that in 6- to 9-year-old group across area and gender between 2002 and 2012 (Table 3).

**Table 2:** Comparison of vitamin A levels in children between 2002 and 2012

Age group (year)	2002						2012					
	Urban			Rural			Urban			Rural		
	Boys	Girls	Total	Boys	Girls	Total	Boys	Girls	Total	Boys	Girls	Total
6–9	0.11 ± 0.03	0.11 ± 0.03	0.11 ± 0.03	0.09 ± 0.02	0.09 ± 0.03	0.09 ± 0.02	0.14 ± 0.02	0.14 ± 0.03	0.14 ± 0.03	0.12 ± 0.02	0.13 ± 0.03	0.13 ± 0.03
10–13	0.13 ± 0.03	0.13 ± 0.04	0.13 ± 0.04^a^	0.11 ± 0.03	0.11 ± 0.03	0.11 ± 0.03^b^	0.15 ± 0.04	0.15 ± 0.03	0.15 ± 0.03^c^	0.14 ± 0.03	0.14 ± 0.03	0.14 ± 0.04^d^
Total	0.12 ± 0.03			0.10 ± 0.03			0.14 ± 0.03^e^			0.13 ± 0.03^f^		
	0.11 ± 0.03						0.14 ± 0.03^g^					

^a^In urban areas at 2002, 10–13 age group compared to 6–9 age group, t = 6.26 *p* = 0.000 statistically significant

^b^In rural areas at 2002, 10–13 age group compared to6–9 age group, t = 12.06 *p* = 0.000 statistically significant

^c^In urban areas at 2012, 10–13 age group compared to 6–9 age group, t = 2.55 *p* = 0.0107 statistically significant

^d^In rural areas at 2012, 10–13 age group compared to 6–9 age group, t = 4.33 *p* = 0.000 statistically significant

^e^In urban areas at 2012 compared to urban in 2002, t = 13.21 *p* = 0.000 statistically significant

^f^In rural areas at 2012compared to rural in 2002, t = 27.96 *p* = 0.000 statistically significant

^g^compared to 2002, t = 39.26 *p* = 0.000 statistically significant

**Table 3:** Comparison of vitamin A deficiency of children between 2002 and 2012(%/n)

Age group (year)	2002						2012					
	Urban			Rural			Urban			Rural		
	Boys	Girls	Total	Boys	Girls	Total	Boys	Girls	Total	Boys	Girls	Total
6–9	5.06/24	4.38/21	4.72/45	13.35/250	12.14/218	12.76/468	11.90/85	9.41/65	10.68/150	9.45/92	8.24/79	8.85/171
10–13	2.64/9	2.55/9	2.59/18^a^	6.64/96	7.38/104	7.00/200^b^	8.61/44	7.66/42	7.64/86^c^	6.80/54	4.08/31	5.47/85^d^
Total	3.83/63			10.24/668			9.33/236^e^			7.34/256^f^		
	8.95/731						8.18/492^g^					

^a^In urban areas at 2002, 10–13 age group compared to 6–9 age group, *x* ^2^ = 4.998 *p* = 0.025 statistically significant

^b^In rural areas at 2002, 10–13 age group compared to6–9 age group, *x* ^2^ = 5.765 *p* = 0.000 statistically significant

^c^In urban areas at 2012, 10–13 age group compared to 6–9 age group, *x* ^2^ = 6.789 *p* = 0.009 statistically significant

^d^In rural areas at 2012, 10–13 age group compared to 6–9 age group, *x* ^2^ = 14.791 *p* = 0.000 statistically significant

^e^In urban areas at 2012 compared to urban in 2002, *x* ^2^ = 45.456 *p* = 0.000 statistically significant

^f^In rural areas at 2012compared to rural in 2002, *x* ^2^ = 33.257 *p* = 0.000 statistically significant

^g^compared to 2002, *x* ^2^ = 2.602 *p* = 0.107 no statistically significant

In 2012, the incidence of marginal VAD in the children was lower than that in 2002 (*x* ^2^ = 861.604, *p* = 0.000). Similar phenomena were also found in across area groups. The incidence of marginal VAD in 10- to 13-year-old group was lower than that in 6- to 9-year-old group across areas and genders between 2002 and 2012. There was no difference in vitamin A status of the children across the area, gender and age groups between 2002 and 2012 (Table 4).

**Table 4:** Comparison of marginal vitamin A deficiency in children between 2002 and 2012(%/n)

Age group (year)	2002						2012					
	Urban			Rural			Urban			Rural		
	Boys	Girls	Total	Boys	Girls	Total	Boys	Girls	Total	Boys	Girls	Total
6–9	32.27/153	32.78/157	32.53/310	51.55/965	50.01/899	50.80/1864	20.72/148	23.30/161	21.99/309	23.82/232	25.86/248	24.83/480
10–13	26.39/90	25.78/91	26.08/181^a^	44.01/636	42.91/605	43.47/1241^b^	22.11/113	16.06/88	17.87/201^c^	20.03/159	22.66/172	21.31/331^d^
Total	29.81/491			49.69/3241			19.80/501^e^			23.26/811 ^f^		
	45.68/3732						21.82/1312^g^					

^a^In urban areas at 2002, 10–13 age group compared to 6–9 age group, *x* ^2^ = 7.980 *p* = 0.005 statistically significant

^b^In rural areas at 2002, 10–13 age group compared to6–9 age group, *x* ^2^ = 34.648 *p* = 0.000 statistically significant

^c^In urban areas at 2012, 10–13 age group compared to 6–9 age group, *x* ^2^ = 94.831 *p* = 0.000 statistically significant

^d^In rural areas at 2012, 10–13 age group compared to 6–9 age group, *x* ^2^ = 5.971 *p* = 0.015 statistically significant

^e^In urban areas at 2012 compared to urban in 2002, *x* ^2^ = 55.192 *p* = 0.000 statistically significant

^f^In rural areas at 2012compared to rural in 2002, *x* ^2^ = 657.579 *p* = 0.000 statistically significant

^g^compared to 2002, *x* ^2^ = 861.604 *p* = 0.000 statistically significant

## Discussion

The main finding of this study indicate that the levels of vitamin A increased with age increase, and VAD and marginal VAD decreased with age increase of the children in China in 2002 and 2012. Our results are consistent with those observed in previous studies [6, 11, 12], suggested greater vulnerability of younger children than older one. It is possibly due to physical growth, adverse effects of virus and bacterial infections, as well as parasitic infections common in younger age group. In addition, 10- to 13-year-old Chinese children was facing the period from primary to junior high school. The majority of parents pay more attention to children’s nutrition, and the greater diversification of dietary pattern observed in those children. It maybe have other reason such as use of one single cut-off point for all age groups, not consideration potentially physiologically lower vitamin A levels in younger children [13]. No significant difference on vitamin A status between boys and girls in this study were consistent with other studies [6, 12]. The reason could be that the Chinese family planning policy that only one child per family, so no matter boys and girls to parents with the same attitude about child’s growth.

During the past decades of rapid socio-economic development in China, urbanization and modernization was great success in improvement of children nutritional status, which resulted in reduced mortality rates, underweight and stunting of children [14]. Our study compared the level of vitamin A of children between 2002 and 2012. The level of vitamin A in 2012 was higher than that in 2002. The reason may be attribute to the promotion of successful agricultural and economic reforms during the past ten years in China. The sustainable food security system kept stable food pricing and food provisions for the nation’s 1.3 billion population, which was able to get the food items essential for health. Increased the income of average family enables parents to provide a variety of food for their children [15]. A significant increase in dietary diversity was observed, and more people consumed food from a larger number of food groups [16]. The intake of vegetables, fruits, cakes, and milk and other animal products like pork, poultry and eggs are increasing [17]. Those dietary food are rich in vitamin A increased the levels of vitamin A of the children at 2012.

There was no significant difference on VAD of the children between 2002 and 2012, but the VAD of the urban children in 2012was higher than that in 2002. A meta-analysis indicated that the prevalence of overweight/obesity has increased significantly among both boys and girls in China from 1981 to 2010. Similar tendency were found from infancy to adolescence in both urban and rural areas. However, the prevalence of overweight/obesity in urban areas was higher than that in rural areas [18]. Several studies found overweight and obese adults and children have lower blood concentrations of vitamins such as vitamin A and minerals compared with those in normal-weight subjects, and β-carotene,as vitamin A precursors,was also declined in overweight and obese adults and children [19–22]. The physiology of micronutrient metabolism was altered in obese and overweight individuals, with greater fat mass resulting in increased sequestering of lipophilic vitamins in adipose tissue [23]. In our future study we should pay more attention to the status of vitamin A in Chinese urban children, especially in overweight and obese children.

In recent years, cases of clinical VAD were considered to represent only the tip of the iceberg, while number of VAD in children was on the decline,with the existence of much larger amount of populations in less advanced (marginal) stages of deficiency, this fact that may also lead to increasing morbidity and mortality among children, newborn babies, women of reproductive age, puerperium and nursing mothers, who traditionally considered to be at risk [12]. Though the prevalence of marginal VAD declined in 2012 (22 %), it was still a moderate public health problem.

The limitation of the present study is that: our study was lack of socioeconomic status, dietary intake, anthropometric data as well as other general information of Chinese children and also our study was not consider about the effect of CRP on the vitamin A status of Chinese school-age children. However, our study was first report that the vitamin A status of Chinese school-age children at national level. In future studies, we will think about more factors which affect the vitamin A status of Chinese school-age children.

## Conclusions

In summary, compared to 2002, the vitamin A status of children was improved in 2012. VAD was still a moderate public health problem in Chinese children. Lower age and in urban of children was easy to occurrence vitamin A deficiency, so in future we should pay more attention to lower age and in urban of children as to prevent vitamin A deficiency occurrence and promote Chinese children’s health.
